# Members of the *Enterobacteriaceae* family produce a diffusible signal factor that modulates enteric pathogen virulence

**DOI:** 10.1128/msphere.00403-26

**Published:** 2026-10-08

**Authors:** Paulina D. Pavinski Bitar, Rimi Chowdhury, Erick Maosa Bosire, Ivan Keresztes, Michael J. Stanhope, Craig Altier

**Affiliations:** 1 Department of Population Medicine and Diagnostic Sciences, College of Veterinary Medicine, Cornell University 43317, Ithaca, New York, USA; 2 Department of Microbiology, College of Arts and Science, Miami University 124575, Oxford, Ohio, USA; 3 Department of Chemistry and Chemical Biology, Cornell University 251791, Ithaca, New York, USA; 4 Department of Public and Ecosystem Health, College of Veterinary Medicine, Cornell University 43317, Ithaca, New York, USA; Meharry Medical College, Nashville, Tennessee, USA

**Keywords:** diffusible signal factor, *cis*-2-tetradecenoic acid, *rpfF*, enteric pathogens, virulence, *Enterobacter*, *Cronobacter*, *Salmonella*, biofilm, motility, quorum sensing

## Abstract

Diffusible signal factors (DSFs) are a gammaproteobacterial family of quorum-sensing signals that mediate the transition between sessile and planktonic lifestyles. In addition to coordinating behavior within producing species, DSFs can be sensed by surrounding organisms, enabling interspecies “eavesdropping.” Several enteric pathogens that do not produce DSFs use these environmental signals to modulate their virulence, allowing them to conserve energy when expression of pathogenesis genes is not advantageous. Although DSFs have been detected in the gut, the microbial origins of these signals remain poorly defined. Here, we show that the ability to produce DSFs is more widespread among *Gammaproteobacteria* than previously appreciated. Using phylogenetic analysis, we identified more than 500 homologs of the DSF synthase RpfF that group into three distinct taxa. Our analysis further revealed evidence of horizontal gene transfer between *Burkholderiales*, a known reservoir of DSF production, and *Enterobacterales*, an order enriched in gut-associated bacteria. We found that *Enterobacterales* members *Cronobacter sakazakii* and *Enterobacter cloacae* produce the DSF *cis*-2-tetradecenoic acid in an *rpfF*-dependent manner. Furthermore, we demonstrated *cis*-2-tetradecenoic acid to be a potent repressor of virulence in the enteric pathogens *Salmonella enterica*, *Shigella flexneri*, and *Vibrio cholerae*. Finally, we have shown that in addition to its role in interspecies signaling, endogenous DSF production reduces biofilm formation and suppresses swarming in *E. cloacae*, while also promoting swimming motility, consistent with a shift to a free-living lifestyle. Together, these findings expand the known diversity of DSF signaling and identify gut-associated bacteria as a source of interspecies cues that shape pathogen behavior.

**IMPORTANCE:**

Many bacterial pathogens rely on tightly regulated gene expression to cause disease, making virulence pathways attractive targets for new therapies. Diffusible signal factors (DSFs) are communication molecules that can suppress the virulence of several enteric pathogens, but their sources in the gut have not been well defined. Here, we identify gut-associated bacteria that produce the DSF molecule *cis*-2-tetradecenoic acid, capable of strongly repressing virulence in multiple enteric pathogens. Additionally, our work substantially expands the distribution of bacteria with the genetic capacity for DSF production, broadening the pool of candidate signal sources and paving the way for future discoveries in microbial communication.

## INTRODUCTION

Quorum sensing is a communication strategy widespread among bacteria that coordinates population-wide behavior through the accumulation of extracellular signals. This mode of cooperation utilizes a variety of signaling molecules to achieve a multicellular lifestyle, facilitating increased virulence (1, 2), greater protection from antibiotics (3, 4) and host defenses (5), and an improved sense of the environment (6). Although often studied as an intraspecies mechanism, quorum-sensing signals can also mediate communication between species. Bacteria can engage in interspecies interactions either by responding to shared signals, such as the conserved autoinducer-2 (7, 8), or by eavesdropping on heterologous quorum-sensing molecules released by neighboring species (9, 10). These interspecies interactions can be mutualistic, for example, by enhancing collective virulence through multi-species biofilms (11, 12) or synergistic induction of virulence factors (13–15). Alternatively, quorum-sensing signals can be deployed antagonistically, as in colonization inhibition, by which the native microbiota prevent transient pathogens from establishing infection (16, 17), or conversely when a bacterium creates a niche for itself by displacing established species (18, 19).

The diffusible signal factor (DSF) family of quorum-sensing signals is especially notable for its ability to span species and even kingdom boundaries, with studies showing effects of bacterial DSFs on the fungus *Candida albicans* as well as on plant and zebrafish hosts (20–24). Produced by a number of gram-negative bacteria, DSFs are primarily a collection of medium- and long-chain fatty acids with a canonical *cis*-2 unsaturation (23, 25, 26). *cis*-2-Unsaturated fatty acids cannot be simply diverted from the standard fatty acid biosynthesis pathway, which terminates in *trans*-2-enoyl intermediates; instead, they are generated by the conserved DSF synthase RpfF, a dual-function acyl-ACP thioesterase and dehydratase (27, 28). To date, *rpfF* has been found in three conserved operon architectures, clustered with related proteins that are involved in signal perception and transduction (23). In the phytopathogenic *Xanthomonas* spp., *Xylella fastidiosa*, and the versatile opportunist *Stenotrophomonas maltophilia*, DSF is sensed by the histidine kinase RpfC, which activates the phosphodiesterase response regulator RpfG to reduce levels of the second messenger cyclic di-GMP and promote the transition from a sessile to a planktonic lifestyle (29–32). In species of the *Burkholderia cepacia* complex, as well as the opportunistic pathogen *Cronobacter turicensis*, DSF signaling disrupts the RpfF/RpfR complex, thereby freeing RpfF for DSF synthesis and activating the phosphodiesterase domain of RpfR to drive cyclic di-GMP degradation (33, 34). By contrast, in *Pseudomonas aeruginosa*, DSF synthesis requires the RpfF homolog DspI (PA0745) and a second enoyl-CoA hydratase, DspII (PA0744) (35, 36), while the associated sensor and phosphodiesterase proteins are encoded at separate genomic loci rather than in a conserved operon (37). Nevertheless, DSF signaling in *P. aeruginosa* ultimately lowers cellular cyclic di-GMP levels, leading to biofilm dispersion, an increase in swarming motility, and an induction of virulence factors and antibiotic resistance genes (15, 38, 39). These *rpfBFCHG*, *rpfFR*, and *dspI-dspII* arrangements represent the three categories of DSF production systems known to date.

Within the gut environment, DSF-mediated interspecies signaling may allow invading pathogens that do not themselves synthesize DSFs to optimize their virulence. Many enteric pathogens deploy an arsenal of virulence factors to overcome colonization resistance, acquire nutrients, and evade the host immune system (40, 41). However, since virulence gene expression is energetically costly, it must be subject to tight regulation (42, 43). A key step of *Salmonella* pathogenesis is the invasion of intestinal epithelial cells, an inflammation-inducing process that alters the gut environment to favor *Salmonella* expansion (44). AraC-type transcriptional regulators HilD, HilC, and RtsA form a feed-forward loop that activates *hilA*, which in turn initiates the invasion gene cascade (45). The master regulator HilD is inhibited by a variety of molecules present in the intestinal environment, including bile acids, short- and long-chain fatty acids, as well as the DSF *cis*-2-hexadecenoic acid (46–50). Additionally, *in vitro* studies have shown that DSFs potently inhibit HilC and RtsA in *Salmonella*, as well as the AraC-type master virulence regulators VirF and ToxT in *Shigella flexneri* and *Vibrio cholerae*, respectively (51–53). While *cis*-2-hexadecenoic acid has been isolated from the gut (54), the diversity of DSFs that may be produced in this environment and the microbial taxa capable of producing them remain largely undefined.

In this study, we identify potential sources of DSFs within the gut microbiota and characterize their impact on enteric pathogen virulence. Using a comparative genomics approach, we identified more than 450 previously unrecognized DSF synthase homologs across the *Proteobacteria* and focused on the gut-associated order *Enterobacterales* for further analysis. Within this order, we show that both the opportunistic pathogen *Cronobacter sakazakii* and the commensal bacterium *Enterobacter cloacae* produce the DSF molecule *cis*-2-tetradecenoic acid. We further show that this signal is a potent repressor of enteric pathogen virulence, and that it modulates biofilm formation and motility in *Enterobacter*. Together, these findings expand the known diversity of DSF signaling molecules and identify gut-associated bacteria as potential contributors to interspecies modulation of virulence.

## RESULTS

### Homologs of the diffusible signal factor synthase RpfF form three distinct taxa

**Fig 1:**
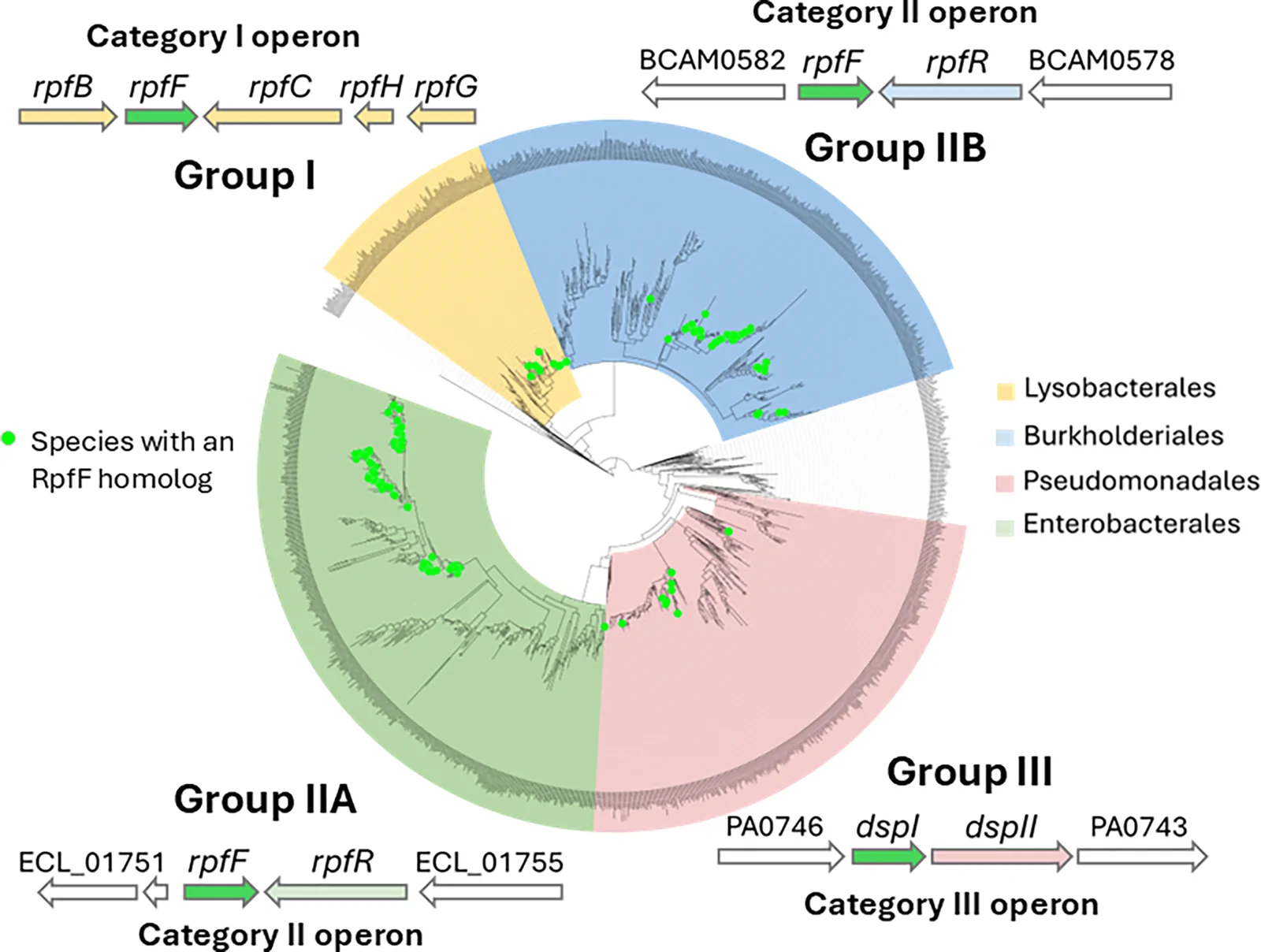
RpfF homologs are widely distributed across *Gammaproteobacteria*. Maximum-likelihood phylogenetic tree based on 16S rRNA gene sequences from representative *Gammaproteobacteria*. Taxonomic groups corresponding to *Lysobacterales*, *Burkholderiales*, *Pseudomonadales*, and *Enterobacterales* are indicated. Species encoding an *rpfF* homolog (≥65% amino acid identity and ≥95% query coverage to a characterized DSF synthase) are marked with green circles. Schematic representations of *rpfF* operon organization from representative genomes are shown adjacent to each group. For clarity, branch support values are not shown.

DSFs, a class of quorum-sensing molecules primarily composed of *cis*-2-unsaturated long-chain fatty acids, have been implicated in interspecies signaling. Several enteric pathogens utilize exogenous DSFs as signals to modulate the expression of their virulence genes. DSFs have been isolated from the murine colon, but organisms responsible for their production in the gut remain largely unidentified. As synthesis of *cis*-2-unsaturated fatty acids is mediated exclusively by the DSF synthase RpfF, we sought to identify gut-associated organisms that contain an RpfF homolog. We used Microbe BLAST to identify proteins that shared at least 65% amino acid identity with published RpfF homologs and for which ≥95% of the query sequence was covered by the alignment, a threshold chosen to enrich for close homologs likely to encode DSF synthases while excluding more distantly related enoyl-CoA hydratase-like proteins. All 502 of the nonredundant homologs identified in our search belonged to the class *Gammaproteobacteria*. A maximum-likelihood tree grouped these 502 proteins into three distinct taxa that correspond to the three well-characterized *rpf* operon categories (Fig. S1 and S2; Table S1; Fig. 1). Group I formed a monophyletic clade consisting exclusively of *Lysobacteraceae* species harboring the *rpfBFCHG* operon characteristic of plant pathogens *Xanthomonas* and *Xylella* spp. Its topology closely mirrors the branching pattern observed in a 16S rRNA phylogeny of *Gammaproteobacteria*, consistent with vertical inheritance (Fig. 1; Fig. S3). In contrast, Group II was polyphyletic and spanned two taxonomically disparate orders, encompassing most of the order *Enterobacterales*, along with distantly related *Burkholderiales* species. This distribution is strongly incongruent with the 16S tree topology and points to a history of horizontal gene transfer of the *rpfFR* operon between these orders. Finally, the third group comprised 142 species, primarily of the order *Pseudomonadales*, that contained the *dspI-dspII* operon previously described in *Pseudomonas aeruginosa* (35, 36). Although the evolutionary relationship among the three groups could not be ascertained, our analysis revealed that RpfF homologs are far more widely distributed among *Gammaproteobacteria* than previously appreciated, a finding that broadens the potential taxonomic scope of DSF producers and supports the feasibility of identifying enteric sources of these signals.

### Enteric pathogen *Cronobacter sakazakii* makes the DSF *cis*-2-tetradecenoic acid capable of repressing *Salmonella* invasion genes

**Fig 2:**
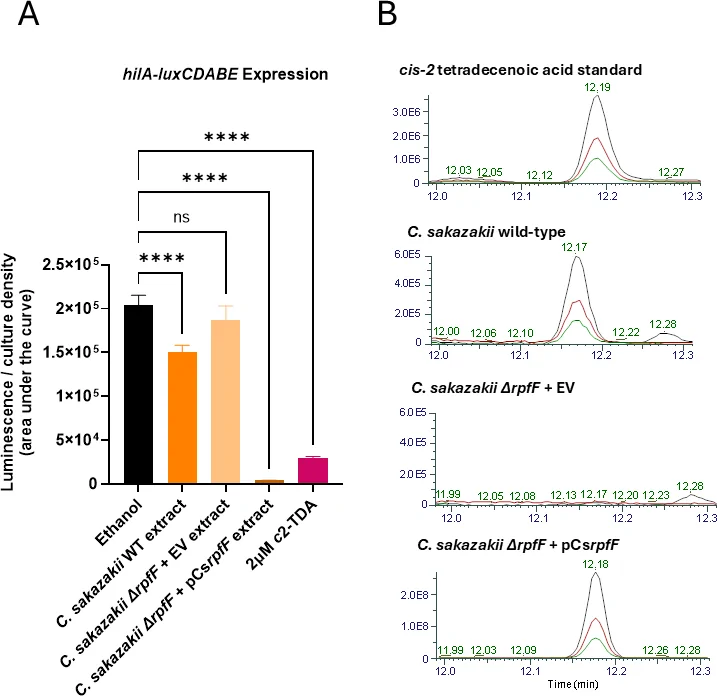
Enteric pathogen *Cronobacter sakazakii* makes the DSF *cis*-2-tetradecenoic acid capable of repressing *Salmonella* invasion genes. (**A**) Effects of *C. sakazakii* fatty acid extracts on *Salmonella* invasion gene expression. Fatty acids were extracted from 12 h cultures of *C. sakazakii* WT, Δ*rpfF* mutant harboring the EV, or the Δ*rpfF* complemented strain (pCs*rpfF*). The extracts were added to cultures of *Salmonella* strains with a *hilA-luxCDABE* transcriptional reporter fusion. A *cis*-2-tetradecenoic acid standard (*c*2-TDA) was included as a control. Luminescence was measured over a 24 h period, normalized to culture density, and summarized as area under the curve (AUC) for each treatment. Bars represent mean ± standard deviation (SD) (*n* = 5); statistical significance was determined by one-way analysis of variance (ANOVA) with Dunnett’s multiple comparisons test. ****, *P* < 0.0001; ns, not significant. (**B**) Extracted ion chromatograms (EICs) of fatty acid samples, highlighting the DSF peak at a retention time of ~12.2 min. Overlaid EICs are shown for *m*/*z* 113.0597 (black), 81.0335 (red), and 143.1067 (green) (±5 ppm). Peak identity was assigned by matching retention time and ion traces to a *cis*-2-tetradecenoic acid standard.

As the order *Enterobacterales* contains multiple gut-associated species, including enteric pathogens like *Salmonella* that are known to sense exogenous DSF-family signals to modulate virulence, we next focused on representative *Enterobacterales* species to determine whether they were capable of DSF synthesis. *Cronobacter sakazakii* is an opportunistic food-borne pathogen of the family *Enterobacteriaceae* (55, 56). Members of the *Cronobacter* genus have been inferred to use *rpfF* to produce a signal capable of exogenous and endogenous DSF-like effects (34), although the specific compound responsible has yet to be isolated. We previously demonstrated that spent supernatant from a *C. sak*a*zakii* culture can repress virulence gene expression in *Vibrio cholerae*, and that deleting *rpfF* alleviates this effect (52). To determine whether *C. sakazakii* is capable of DSF synthesis, we isolated and identified the compounds responsible for these repressive effects by testing organic extracts from cultures of wild-type (WT) and Δ*rpfF C. sakazakii* strains for their ability to repress the expression of *Salmonella* invasion genes known to be sensitive to DSFs (49). Using a *hilA-luxCDABE* reporter fusion, we found that extracts from the wild-type strain significantly repressed *hilA* expression, while extracts from the Δ*rpfF* mutant failed to repress *hilA* (Fig. 2A). Complementation with a plasmid-borne *rpfF* resulted in 48-fold repression relative to the empty vector (EV) control, restoring and exceeding the wild-type phenotype, consistent with overexpression from a multicopy plasmid. Subsequent gas chromatography–mass spectrometry (GC–MS) analysis identified the repressive signal produced by the wild-type and complemented *C. sakazakii* strains as *cis*-2-tetradecenoic acid (Fig. 2B), a 14-carbon unbranched fatty acid with the characteristic unsaturation at the second carbon that has previously been identified to function as a DSF in *X. fastidiosa* (31). GC–MS spectra of the *rpfF* knockout strain lacked peaks associated with DSFs, including *cis*-2-tetradecenoic acid, suggesting that *rpfF* is necessary for proper DSF production in this strain. Thus, *C. sakazakii* provides direct evidence that a functional *rpfFR* operon can operate in *Enterobacterales* and represents the first report of *cis*-2-tetradecenoic acid signaling in this order, positioning it as a bona fide reservoir of DSF-mediated interbacterial signaling.

### RpfF of the healthy gut constituent *Enterobacter cloacae* generates the DSF *cis*-2-tetradecenoic acid

**Fig 3:**
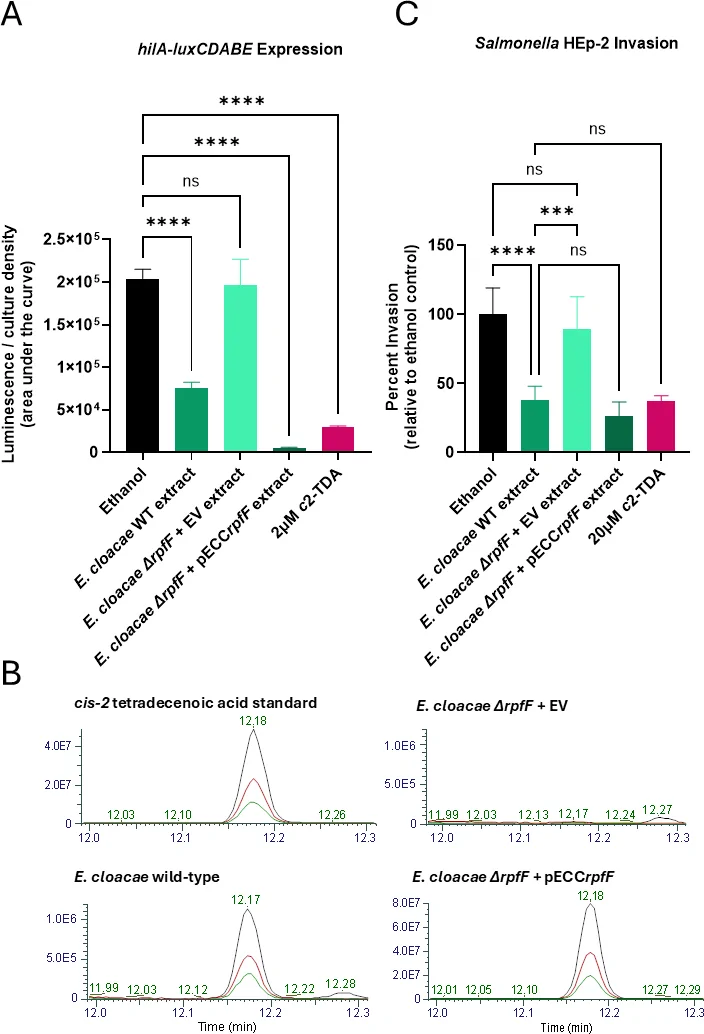
*Enterobacter cloacae* produces *rpfF*-dependent DSF that represses *Salmonella* invasion gene expression and host cell invasion. (**A**) Effects of *E. cloacae* fatty acid extracts on *Salmonella* invasion gene expression. Fatty acids were extracted from 12 h cultures of *E. cloacae* WT, the Δ*rpfF* mutant carrying an EV, or the Δ*rpfF* complemented strain (pECC*rpfF*). Extracts were added to cultures of *Salmonella* strains carrying a *hilA-luxCDABE* transcriptional reporter fusion. A *cis*-2-tetradecenoic acid standard (*c*2-TDA) was included as a control. Luminescence was measured over a 24 h period, normalized to culture density, and summarized as AUC for each treatment. Bars represent mean ± SD (*n* = 5); statistical significance was determined by one-way ANOVA with Dunnett’s multiple comparisons test. ****, *P* < 0.0001; ns, not significant. (**B**) Extracted ion chromatograms (EICs) of fatty acid extracts highlighting the DSF peak at a retention time of ~12.2 min. Overlaid EICs are shown for *m*/*z* 113.0597 (black), 81.0335 (red), and 143.1067 (green) (±5 ppm). Peak identity was assigned by matching retention time and ion traces to a *cis*-2-tetradecenoic acid standard. (**C**) Effects of *E. cloacae* fatty acid extracts on *Salmonella* invasion of HEp-2 cells. *Salmonella* cultures were treated with the indicated extracts or *c*2-TDA prior to infection. Invasion was quantified using a gentamicin protection assay and expressed as a percentage of the solvent (ethanol)-treated control. Bars represent mean ± SD (*n* = 5); statistical significance was determined by one-way ANOVA followed by Tukey’s multiple comparisons test. ****, *P* < 0.0001; ***, *P* < 0.001; ns, not significant.

Members of the family *Enterobacteriaceae* are a small but integral part of a healthy human gut microbiome, aiding in vitamin production, pathogen resistance, and the creation of an anaerobic environment (57). With the exception of *Escherichia coli*, species of the *Enterobacter* genus are the most common *Enterobacteriaceae* found in the normal gut (58–60). As we found the *rpfFR* operon to be conserved among the *Enterobacter* genus, we next sought to determine if a commensal *Enterobacter* strain produced compounds that could repress *Salmonella* invasion gene expression. We thus grew the *hilA-luxCDABE* reporter strain in medium supplemented with organic extracts from either wild-type or Δ*rpfF E. cloacae* culture supernatants. Addition of the wild-type *E. cloacae* extract led to a 2.7-fold reduction in *hilA* expression, while extracts from the Δ*rpfF* mutant did not significantly alter *hilA* expression compared to the solvent control (Fig. 3A). Once more, complementation restored and exceeded wild-type repression levels, consistent with the hypothesis that a repressive extracellular signal is produced by *E. cloacae* in an *rpfF*-dependent manner. Next, we used GC–MS to identify the repressive compound produced by *E. cloacae rpfF*, finding it again to be the DSF *cis*-2-tetradecenoic acid (Fig. 3B). Finally, we assessed the effect of these extracts on the ability of *Salmonella* to invade host cells, a critical step in virulence and colonization. Using a gentamicin protection assay, we evaluated the impact of exposure to the extracts on the invasion of HEp-2 cells *in vitro*. Extracts from wild-type *E. cloacae* significantly reduced invasion to 38% of untreated *Salmonella* levels, matching the effect of 20 μM *cis*-2-tetradecenoic acid, whereas extracts from the Δ*rpfF* mutant had no effect (Fig. 3C). Complementation restored the repressive phenotype to above wild-type levels, reducing invasion to 27% of untreated levels. Taken together, our findings identify the healthy gut constituent *E. cloacae* as a potential source of repressive DSF signals that may serve to inhibit *Salmonella* invasion genes in the enteric environment.

### *cis*-2-Tetradecenoic acid is a potent repressor of enteric pathogen virulence

**Fig 4:**
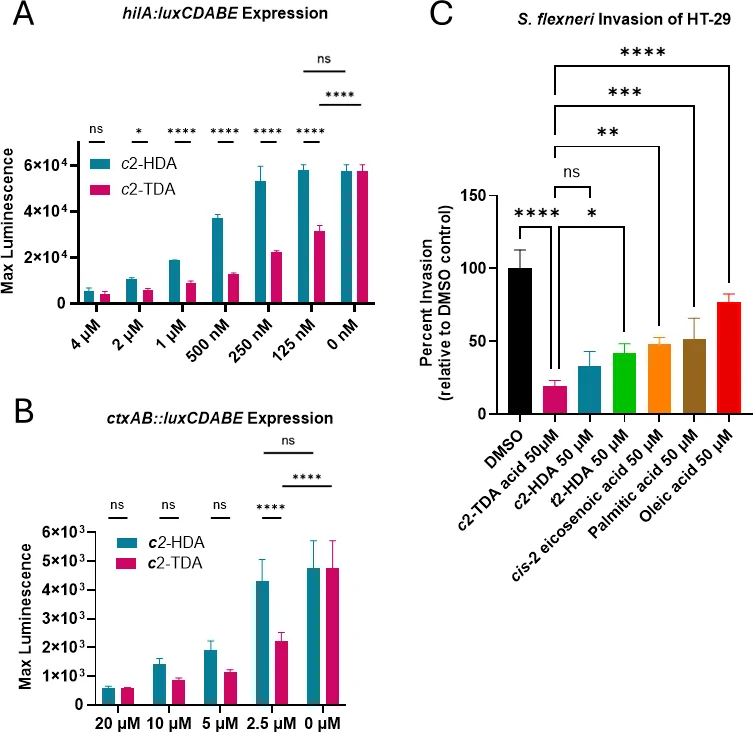
*cis*-2-tetradecenoic acid is a potent repressor of enteric pathogen virulence. (**A**) Concentration-dependent effects of *cis*-2-tetradecenoic acid (*c*2-TDA) and *cis*-2-hexadecenoic acid (*c*2-HDA) on *Salmonella* invasion gene expression. *Salmonella* strains carrying a *hilA-luxCDABE* transcriptional reporter were treated with the indicated concentrations of each compound. Luminescence was measured over a 24 h period and normalized to culture density. Bars represent mean peak normalized luminescence ± SD (*n* = 5); statistical significance was determined by two-way ANOVA with Šídák’s multiple comparisons test for pairwise comparisons and Dunnett’s test for comparisons to the untreated (0 nM) control. ****, *P* < 0.0001; *, *P* < 0.05; ns, not significant. (**B**) Effects of *c*2-TDA and *c*2-HDA on *Vibrio cholerae* virulence gene expression. *V. cholerae* strains carrying a *ctxAB-luxCDABE* transcriptional reporter were treated with the indicated concentrations of each compound. Luminescence was measured and processed as in panel **A**. Bars represent mean peak normalized luminescence ± SD (*n* = 5); statistical significance was determined by two-way ANOVA with Šídák’s multiple comparisons test for pairwise comparisons and Dunnett’s test for comparisons to the untreated (0 nM) control. ****, *P* < 0.0001; ns, not significant. (**C**) Effects of fatty acids on *S. flexneri* invasion of HT-29 cells. *S. flexneri* cultures were treated with the indicated fatty acids prior to infection. Invasion was quantified using a gentamicin protection assay and expressed as a percentage of solvent (DMSO)-treated control levels. Bars represent mean ± SD (*n* = 4); statistical significance was determined by one-way ANOVA followed by Šídák’s multiple comparisons test. ****, *P* < 0.0001; ***, *P* < 0.001; **, *P* < 0.01; *, *P* < 0.05; ns, not significant. *trans*-2-Hexadecenoic acid is abbreviated as *t*2-HDA.

We have previously shown that several enteric pathogens within the class *Gammaproteobacteria* exhibit repressed virulence when exposed to DSFs, particularly the potent repressor *cis*-2-hexadecenoic acid (52–54). The DSF *cis*-2-tetradecenoic acid has previously been isolated from the grape pathogen *X. fastidiosa* (31) of the order *Lysobacterales*, but its effects on enteric pathogen virulence have not been explored. We therefore sought to quantify the ability of *cis*-2-tetradecenoic acid to attenuate virulence in DSF-responsive enteric pathogens and to compare its activity with that of *cis*-2-hexadecenoic acid. First, we examined the effects of both signals across a range of concentrations on the expression of invasion genes by *Salmonella* using the *hilA-luxCDABE* reporter strain. Both DSFs repressed the reporter in a concentration-dependent manner; however, *cis*-2-tetradecenoic acid consistently produced greater repression at each concentration tested (Fig. 4A). Notably, its effect remained significant at the lowest concentration used (125 nM), whereas repression by *cis*-2-hexadecenoic acid was no longer significant below 500 nM.

Next, we compared the effects of the two compounds on the expression of *V. cholerae* virulence genes using a *ctxAB-luxCDABE* fusion. Because *ctxAB* encodes the cholera toxin and is directly controlled by the AraC-type transcriptional regulator ToxT, this reporter serves as a sensitive indicator of virulence regulation in this species (52, 61). While both DSFs showed a 3.6-fold repression at high concentrations (20 µM), *cis*-2-tetradecenoic acid exhibited greater activity at lower concentrations, remaining significantly repressive at 2.5 µM, whereas *cis*-2-hexadecenoic acid lost significant activity at this dose (Fig. 4B). We have previously shown that DSFs strongly impair the ability of *Shigella flexneri* to invade intestinal epithelial cells by acting directly on VirF, the master regulator of *Shigella* virulence genes (53). We therefore compared the effect of 50 µM *cis*-2-tetradecenoic acid on *S. flexneri* invasion to equimolar concentrations of a panel of repressive fatty acids using the HT-29 cell line. c*is*-2-Tetradecenoic acid treatment led to an approximately fivefold reduction in invasion, which was significantly greater than seen for treatments with oleic, palmitic, *cis*-2-eicosenoic, and *trans*-2-hexadecenoic acids, but not significantly different from that of *cis*-2-hexadecenoic acid (Fig. 4C). Collectively, these results establish *cis*-2-tetradecenoic acid as one of the most potent repressors of enteric pathogen virulence identified thus far.

### Endogenous DSF *cis*-2-tetradecenoic acid disrupts biofilm and encourages swimming over swarming motility in *Enterobacter cloacae*

**Fig 5:**
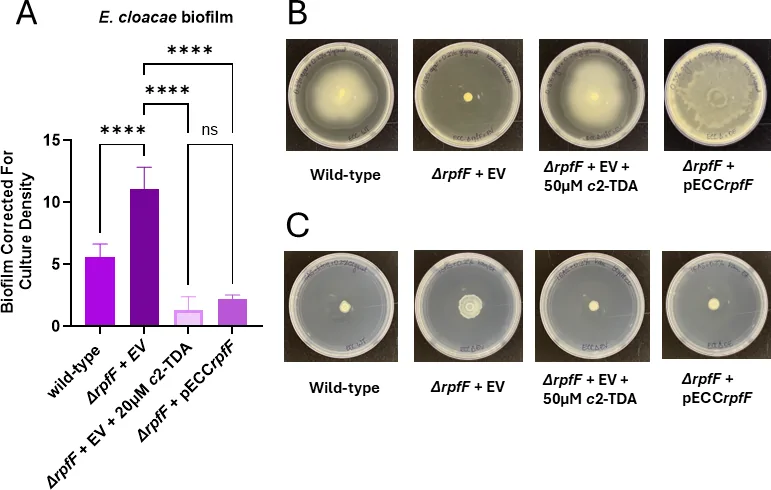
Endogenous DSF *cis*-2-tetradecenoic acid disrupts biofilm and encourages swimming over swarming motility in *Enterobacter cloacae*. (**A**) Effects of endogenous DSF production on *E. cloacae* biofilm formation. WT, Δ*rpfF* mutant, Δ*rpfF* supplemented with *cis*-2-tetradecenoic acid (*c*2-TDA), and the complemented Δ*rpfF* strain (pECC*rpfF*) were grown in M9 minimal medium supplemented with 0.4% glucose. Surface-associated biomass was quantified by crystal violet staining and normalized to culture density (A550/A600). Bars represent mean ± SD (*n* = 8); statistical significance was determined by one-way ANOVA followed by Šídák’s multiple comparisons test. ****, *P* < 0.0001; ns, not significant. (**B**) Effects of DSF on swimming motility in *E. cloacae*. Wild-type, Δ*rpfF* mutant, complemented, and *c*2-TDA-supplemented strains were inoculated onto 0.3% agar plates and incubated overnight under humid conditions. Swimming motility was assessed by radial expansion from the site of inoculation. Representative images are shown. (**C**) Effects of DSF on swarming motility in *E. cloacae*. Strains were inoculated onto 0.5% agar plates supplemented with glucose and incubated overnight. Surface expansion was assessed by the formation of a swarming halo. Representative images are shown.

Organisms that are known to produce DSFs have been shown to use this quorum-sensing signal to switch between a sessile and a planktonic lifestyle, disrupting biofilms and aiding in dissemination in some species (34, 37, 62, 63), while increasing attachment and swarming motility in others (31, 64, 65). Recent studies suggest that the two functions are not mutually exclusive but rather dose-, condition-, or growth-dependent (64, 66, 67). To assess the effect of endogenous DSF on *E. cloacae* biofilm formation, we compared the ability of wild-type and Δ*rpfF* mutant *E. cloacae* strains to produce surface-associated biomass using a crystal violet assay. We observed a twofold increase in biofilm in the absence of *rpfF*, suggesting that DSF plays a biofilm-disrupting role under the conditions tested (Fig. 5A). Consistent with this interpretation, supplementation of the *ΔrpfF* mutant with 20 µM *cis*-2-tetradecenoic acid led to an 8.5-fold reduction in biofilm. Likewise, complementation of the Δ*rpfF* mutant led to a fivefold decrease in biofilm formation, further supporting a biofilm-inhibitory role of DSF. Because DSF can also influence motility, we next examined whether endogenous DSF shifts *E. cloacae* from surface-associated swarming toward planktonic swimming. When incubated on swimming-conducive 0.3% agar plates, wild-type *E. cloacae* formed a distinct pattern of radial expansion, while the Δ*rpfF* mutant remained confined to the site of inoculation (Fig. 5B). Complementation or supplementation with 50 µM exogenous *cis*-2-tetradecenoic acid restored the swimming phenotype. In contrast, when inoculated on 0.5% agar plates supplemented with glucose, conditions known to encourage swarming, the wild-type strain exhibited little to no surface expansion, whereas the Δ*rpfF* mutant formed a swarming halo surrounding the inoculation site (Fig. 5C). Both complementation and exogenous DSF inhibited swarming expansion. Taken together, these results identify DSF as a key regulator of the transition between sessile and motile states in *E. cloacae*, inhibiting biofilm formation and swarming while favoring swimming motility under the conditions tested.

## DISCUSSION

Quorum-sensing signals of the DSF family exhibit broad interspecies and interkingdom signaling activity, while also being exploited by enteric pathogens to modulate virulence independent of population density. In this work, we identified more than 450 species harboring a DSF synthase homolog and showed that gut-associated *Enterobacterales* species *Cronobacter sakazakii* and *Enterobacter cloacae* make the DSF *cis*-2-tetradecenoic acid, a potent repressor of enteric pathogen virulence. Our bioinformatic survey identified RpfF homologs exclusively among the *Gammaproteobacteria*, suggesting a shared evolutionary origin in that class. As our search employed a conservative ≥65% amino acid identity threshold, it likely underestimates the full diversity of RpfF-like enzymes. Expanding the data set to include more divergent homologs may provide additional evolutionary insight, but given the substantial homology between RpfF and the enoyl-CoA hydratase/isomerase family of proteins, future studies will first need to determine whether divergent candidates are capable of DSF production.

By contrast, the evolutionary history within individual clades could be more readily resolved. For instance, within the *Enterobacterales*, our analysis suggests that the DSF synthase *rpfF* was acquired in an operon with the phosphodiesterase *rpfR* by the common ancestor of the *Enterobacteriaceae*, *Erwiniaceae*, and *Yersiniaceae* lineages, followed by a subsequent loss along the branch leading to the *Pectobacteriaceae* clade. Notably, the clade comprising the enteric pathogens *Salmonella*, *Citrobacter*, *Escherichia coli*, and *Shigella* lost *rpfF* while retaining *rpfR*. In these species, the *rpfR* homolog, more commonly referred to as *pdeR* or *yciR*, functions as a phosphodiesterase that reduces cyclic di-GMP levels, thereby promoting curli biogenesis and biofilm formation through the *csgD* regulatory cascade (68–70). An important outstanding question is whether these RpfR homologs retain the capacity to sense and respond to DSF signals despite the loss of endogenous DSF production.

In addition, we have shown that these and other enteric pathogens modulate their virulence in response to exogenous DSF signals through direct interaction with the AraC-type transcriptional regulators that control vital virulence functions (49, 51, 53, 54, 71). This regulation is distinct from the mechanism by which DSF producers sense and respond to their own signals. Rather than altering cyclic di-GMP levels, DSF binding to AraC-type regulators disrupts their DNA binding capacity, preventing their association with target gene promoters. This intricate system of control suggests coevolution between enteric pathogens and the native microbiota of the gut. However, as *Cronobacter* and *Enterobacter* are likely transient members of the gut microbiota rather than stable colonizers, it is unlikely that enteric pathogens evolved to respond specifically to their signals. Instead, AraC-type regulators in these pathogens may have evolved to respond to a broad range of DSF-family signals rather than a single molecule. Alternatively, they may use as their primary ligand the DSF *cis*-2-hexadecenoic acid, which has been detected in the gut and differs from *cis*-2-tetradecenoic acid only in chain length (54). Nevertheless, even if *cis*-2-tetradecenoic acid is not the primary physiological signal, its potent repression of pathogen virulence suggests potential therapeutic applications.

## MATERIALS AND METHODS

### Identification and phylogenetic analysis of RpfF homologs

RpfF homologs were identified using Microbial Nucleotide BLAST by querying amino acid DSF synthase sequences from *Xanthomonas campestris*, *Xylella fastidiosa*, *Stenotrophomonas maltophilia*, *Pseudomonas aeruginosa*, *Burkholderia cenocepacia*, and *Cronobacter turicensis* against a translated database of representative genomes. Proteins sharing ≥65% amino acid identity and ≥95% query coverage were retained to enrich for close homologs while excluding more distantly related enoyl-CoA hydratase-like proteins. Redundant sequences were removed to generate a nonredundant data set. The resulting 502 amino acid sequences were aligned using MAFFT with default parameters (72). For comparison, a reference phylogeny of *Gammaproteobacteria* was constructed using 16S rRNA gene sequences obtained from the SILVA SSURef NR99 database (73), which was parsed using Biostrings in R to extract representative sequences from ~1,300 species (74). These sequences were aligned using MAFFT, and the resulting alignment was inspected and manually curated using Geneious. Maximum-likelihood phylogenetic trees for both data sets were constructed using IQ-TREE v3 with automatic model selection (ModelFinder) (75, 76). Branch support was assessed using ultrafast bootstrap approximation (1,000 replicates) and SH-like approximate likelihood ratio test (SH-aLRT; 1,000 replicates). Trees were visualized using the Interactive Tree of Life (77). Taxa were defined based on phylogenetic topology and further characterized by examining the genomic context of *rpfF* homologs to determine operon organization.

### Bacterial strains, cell lines, and growth conditions

Bacterial strains and plasmids used in this study are listed in Table 1. *Enterobacter cloacae*, *Cronobacter sakazakii*, *Salmonella enterica* serovar Typhimurium, *Shigella flexneri* 2547T, and *Vibrio cholerae* strains were routinely grown in LB broth (10 g tryptone, 5 g yeast extract, and 5 g NaCl per liter) or on LB agar plates supplemented with 1.5% agar at 37°C unless otherwise indicated. Liquid cultures were grown with shaking at 200 rpm. Where appropriate, antibiotics were added at the following concentrations: ampicillin (100 µg/mL), kanamycin (50 µg/mL), and chloramphenicol (20 µg/mL). HEp-2 and HT-29 cell lines were maintained in high-glucose Dulbecco's modified Eagle medium (HyClone) supplemented with 10% fetal bovine serum (Gibco) at 37°C with 5% CO_2_. Fatty acids were obtained from Cayman Chemical unless otherwise indicated. *cis*-2-Tetradecenoic and *cis*-2-hexadecenoic acids were purchased from Larodan (Solna, Sweden). Compounds were dissolved in ethanol or dimethyl sulfoxide (DMSO) to prepare stock solutions. For experimental treatments, fatty acids were added to cultures at the indicated final concentrations, and control cultures received an equivalent volume of solvent.

**TABLE 1:** Strains and plasmids used in the study

Material	Description	Source
Strain		
CA32	*Salmonella enterica* serovar Typhimurium 14028s	ATCC
CA5399	*S. enterica*, p*hilA-luxCDABE-cat*	71
CA216	*Shigella flexneri* strain 2457T	Orndorff lab
CA5588	*Vibrio cholerae*, *lacZ*::*ctxAB-luxCDABE*	52
CA5823	*Cronobacter sakazakii* strain ATCC 29544	ATCC
CA6216	*C. sakazakii* Δ*rpfF +* pACYC177	This study
CA6209	*C. sakazakii* Δ*rpfF* + pCA331 (pCs*rpfF*)	This study
CA5419	*Enterobacter cloacae* subsp. *cloacae* strain ATCC 13047	ATCC
CA6217	*E. cloacae* Δ*rpfF +* pACYC177	This study
CA6212	*E. cloacae* Δ*rpfF +* pCA332 (pECC*rpfF*)	This study
Plasmid		
pACYC177	EV used in this study	78
pCA331	pCs*rpfF—C. sakazakii rpfF* complement plasmid	This study
pCA332	pECC*rpfF—E. cloacae rpfF* complement plasmid	This study

### Construction of *rpfF* deletion mutants

*C. sakazakii* and *E. cloacae* Δ*rpfF* mutants were generated using the λ Red recombinase method as previously described (79). Briefly, the *rpfF* coding sequence was replaced with a kanamycin resistance cassette amplified from plasmid pKD4 using PCR primers containing homology to the flanking regions of *rpfF*. Recombination was carried out in strains expressing the λ Red recombinase from plasmids pKD46 and pKD119, respectively, and recombinant clones were selected on LB agar containing kanamycin. The kanamycin resistance cassette was subsequently removed using the FLP recombinase encoded by plasmid pCP20 to generate unmarked deletion mutants. Successful deletion of *rpfF* was confirmed by PCR.

### Construction of complementation plasmids

Complementation plasmids were constructed by cloning the *rpfF* coding sequences, including the native Shine-Dalgarno regions, from *C. sakazakii* and *E. cloacae* into plasmid pACYC177 using ApaLI and ScaI restriction sites, disrupting the ampicillin resistance gene. Primers used for amplification are listed in Table 2. Recombinant plasmids were transformed into NEB 5-alpha *E. coli* (cat. C2987H) and selected based on loss of ampicillin resistance and retention of kanamycin resistance. Plasmid sequences were verified by sequencing (Plasmidsaurus). Confirmed constructs were introduced into the corresponding Δ*rpfF* mutants for complementation experiments.

**TABLE 2:** Primers used in this study

Primer name	Primer sequence
Cs-*rpfF*-cam-DW-F	5′-CTGATCCTGCGCATAACGCGTTAACTTACATGAG GAAAGACTCCATGGTCCATATGAATATCCTCCTTAG TTC-3′
Cs-*rpfF*-cam-DW-R	5′-CGGGAAACAGACGCCGTAACCGGCGTCTGAGG AATGGCCGGATTGTGTAGGCTGGAGCTGCTTC-3′
camCHK-F	5′-TTCTTGCCCGCCTGATGAAT-3′
Cs-CHK-R	5′-AAGCCCCATTGATGTCGAGATCAC-3′
ECC-*rpfF*-kan-DW-F	5′-ATTAGTCTAAAACTGCGCGCAAGGCGCTTTTTTATA TGAGGAATTAGACTGATTGTGTAGGCTGGAGCTGCT TC-3′
ECC-*rpfF*-kan-DW-R	5′-GCTGCCGCATTCGAGCGATGGCTAAAGCGATATC AGGCGCGTAAACAGCACCATGGTCCATATGAATATC CTCCTTAGTTC-3′
kanCHK-F	5′-TGGCTGCTATTGGGCGAA-3′
ECC-CHK-R	5′-AACGGAAAGCTGCCTCATTGAAAA-3′
Cs*rpfF*-ApaLI-F	5′-CATTGCAGTGCACCATAGGAGGAAAGACTATGT CAGTATTCAACCAAT-3′
Cs*rpfF*-ScaI-R	5′-TATGGCAAGTACTGCCGTTAGCCTGCCTTAC GCAGGG-3′
ECC*rpfF*-ApaLI-F	5′-CATTGCAGTGCACCATAGATGAGGAATTAGACTAT GACAGTTATCAATCAGGC-3′
ECC*rpfF*-ScaI-R	5′-TATGGCAAGTACTGCACTAACTGGCTTTGCGTA AACCCG-3′

### Fatty acid extraction

Fatty acids were extracted from culture supernatants of *C. sakazakii* and *E. cloacae*. Briefly, 50 mL cultures were grown for 12 h in LB broth supplemented with 0.2% glycerol and antibiotics as required for plasmid maintenance. Addition of glycerol consistently enhanced DSF production under these conditions. Cultures were pelleted by centrifugation at 7,500 × *g* for 30 min, and the spent supernatants were collected.

Organic compounds were extracted with 2 volumes of ethyl acetate and mixed thoroughly. After phase separation, the upper phase was collected and evaporated under airflow. The resulting residue was resuspended in 300 µL ethanol and used for GC–MS analysis to identify repressive compounds. To enrich for fatty acids, crude extracts were further processed using a modified Dole extraction method (80, 81). The 300 µL ethanolic extract was combined with 5 parts Dole reagent (isopropanol:heptane:1 N HCl, 40:10:1, vol/vol), followed by addition of 2 parts water and 6 parts heptane. Samples were mixed thoroughly and allowed to separate into phases. The upper organic phase was collected, evaporated under airflow, and resuspended in 200 µL ethanol. These enriched extracts were used in all gene expression and functional assays.

### Gene expression/luciferase assays

Bacterial strains carrying *luxCDABE* transcriptional reporter fusions, either on plasmids or integrated into the chromosome, were cultured overnight in LB supplemented with the appropriate antibiotics. For *Salmonella* reporter assays, the overnight cultures were grown in the presence of 1 mM nonanoic acid to suppress basal invasion gene expression and reduce background luminescence. Overnight cultures were washed with PBS and resuspended in the appropriate media to a starting optical density at 600 nm (OD600) of 0.02. *Salmonella* assays were carried out in LB buffered with 100 mM MOPS (3-morpholinopropane-1-sulfonic acid, pH 6.7). *V. cholerae* reporter assays were done in AKI medium (5 g NaCl, 3 g NaHCO_3_, 4 g yeast extract, and 15 g peptone per liter) prepared as previously (52, 82). Cultures were dispensed into 96-well plates (150 µL per well) containing solvent control, 10 µL fatty acid extracts, or purified compounds at the concentrations indicated. Plates were incubated at 37°C, and luminescence and absorbance (OD600) were measured at 30 min intervals for 24 h using a BioTek Synergy H1 microplate reader. Luminescence values were normalized to culture density, and AUC or peak normalized luminescence values were calculated as indicated.

### Invasion assays

Invasion was assessed using a gentamicin protection assay as previously described with some modifications (83). HEp-2 and HT-29 epithelial cell monolayers were seeded at 1 × 10^5^ cells per well and allowed to adhere overnight. *Salmonella* and *S. flexneri* strains were grown overnight in LB or tryptic soy broth, respectively, at 37°C with aeration. Cultures were pre-incubated with the indicated treatments prior to infection. Fatty acid standards were added at the concentrations described, while fatty acid extracts were added at a 1:100 (vol/vol) final dilution. Control samples received an equivalent volume of solvent. Bacterial densities were measured, and cultures were adjusted in PBS for infection. *Salmonella* was added at 1 × 10^6^ colony-forming units (CFU) per well (MOI, 10), while *S. flexneri* was added at 1 × 10^7^ CFU per well (MOI, 100). Plates were centrifuged at 500 × *g* for 10 min to synchronize invasion and incubated at 37°C with 5% CO_2_ for 1 h (*Salmonella*) or 30 min (*S. flexneri*). Monolayers were washed with PBS and incubated with media containing 200 µg/mL gentamicin for 1 h. After incubation, cells were washed with PBS and lysed with 0.1% Triton X-100 to recover intracellular bacteria. Serial dilutions of lysates and inocula were plated on appropriate agar (LB agar for *Salmonella* and tryptic soy agar for *S. flexneri*) to enumerate CFU. Invasion was calculated as the proportion of recovered bacteria relative to the inoculum and normalized to untreated control levels.

### Biofilm assay

Biofilm formation was assessed using a crystal violet staining assay as described previously with modifications (34, 84). *E. cloacae* wild-type, Δ*rpfF* mutant, and complemented strains were grown overnight in LB supplemented with appropriate antibiotics. Cultures were diluted 1:100 into M9 minimal medium supplemented with 0.4% glucose, and fatty acids or the solvent control (ethanol) were added as indicated. Aliquots were dispensed into clear, flat-bottom 96-well plates and incubated statically at 30°C overnight. After incubation, culture density (OD600) was measured, and wells were washed to remove non-adherent cells. Attached cells were stained with 0.1% crystal violet, then washed and allowed to dry completely. To quantify biofilm biomass, crystal violet was solubilized with 30% acetic acid, and absorbance was measured at 550 nm (A550). Biofilm formation was expressed as A550 normalized to culture density.

### Motility assays

Swimming and swarming motility assays were performed as described previously with modifications (85). For swimming assays, LB plates containing 0.3% agar supplemented with 0.2% glycerol were used. For swarming assays, plates contained 0.5% each of tryptone, NaCl, glucose, and agar. Plates were dried with lids partially open in a biosafety cabinet for 15 min prior to inoculation to avoid over-drying, which can impact motility. Overnight cultures of *E. cloacae* wild-type, Δ*rpfF* mutant, and complemented strains were inoculated (10 µL) at the center of each plate. Following inoculation, plates were allowed to rest for 20 min with lids closed for swimming assays or 15 min with lids open for swarming assays. Swimming plates were incubated upright at 37°C overnight in a humid chamber to prevent drying, while swarming plates were incubated inverted at 30°C overnight. Motility was assessed by radial expansion from the site of inoculation.

### GC–MS

Fatty acid extracts were analyzed by GC–MS to identify DSF species. Extracts (25 µL in ethanol) were derivatized by esterification overnight in 100 µL of a 1:1 mixture of hexane and 3-(trifluoromethyl)phenyltrimethylammonium hydroxide (TCI Chemicals). Samples were analyzed using a Thermo Scientific Exactive GC-Orbitrap GC–MS/MS system equipped with an Agilent Ultra 2 column (19091B-102). Fatty acid methyl esters were injected in split mode (50:1) with an injector temperature of 250°C. The oven temperature program was as follows: 100°C for 2 min, increase at 15°C/min to 150°C and hold for 4 min, then increase at 10°C/min to 280°C and hold for the remainder of the 30 min run. The mass spectrometer was operated in electron ionization mode at 70 eV. The ion source temperature was set to 280°C, and the transfer line temperature was maintained at 250°C. Data were acquired in full scan mode (*m*/*z*, 35–550) at a resolving power of 60,000 (at *m*/*z*, 200). Compound identity was determined by comparison of retention times and mass spectra to an authentic *cis*-2-tetradecenoic acid standard (Larodan). Spectra were analyzed using FreeStyle (version 1.8 SP).

### Statistical analysis

All statistical analyses were performed using GraphPad Prism (version 11.0.1). Data are presented as mean ± SD, unless otherwise indicated. Experiments were performed with 4–8 biological replicates (typically *n* = 5), as specified in the figure legends. Statistical significance was determined using one-way or two-way ANOVA, as appropriate for each experiment, followed by multiple comparisons tests including Dunnett’s, Tukey’s, or Šídák’s tests as specified in the figure legends. Differences were considered statistically significant at *P* < 0.05. Significance levels are denoted as follows: *, *P* < 0.05; **, *P* < 0.01; ***, *P* < 0.001; and ****, *P* < 0.0001.

## SUPPLEMENTAL MATERIAL

Figure S1 msphere.00403-26-s0001.tif ML phylogenetic tree of RpfF homologs.

Figure S2 msphere.00403-26-s0002.pdf High-resolution phylogenetic tree based on RpfF amino acid sequences.

Figure S3 msphere.00403-26-s0003.pdf RpfF homologue presence mapped onto a high resolution 16S rRNA tree.

Legends msphere.00403-26-s0004.docx Supplemental figure legends.

Table S1 msphere.00403-26-s0005.csv *rpfF* homologue taxonomy and sequence data.

ASM does not own the copyrights to Supplemental Material that may be linked to, or accessed through, an article. The authors have granted ASM a non-exclusive, world-wide license to publish the Supplemental Material files. Please contact the corresponding author directly for reuse.

## References

[1] Rutherford ST, Bassler BL. 2012. Bacterial quorum sensing: its role in virulence and possibilities for its control. Cold Spring Harb Perspect Med 2:a012427. doi:10.1101/cshperspect.a012427 PMC3543102 23125205

[2] Zhu J, Miller MB, Vance RE, Dziejman M, Bassler BL, Mekalanos JJ. 2002. Quorum-sensing regulators control virulence gene expression in *Vibrio cholerae*. Proc Natl Acad Sci USA 99:3129–3134. doi:10.1073/pnas.052694299 PMC122484 11854465

[3] Zhao X, Yu Z, Ding T. 2020. Quorum-sensing regulation of antimicrobial resistance in bacteria. Microorganisms 8:425. doi:10.3390/microorganisms8030425 PMC7143945 32192182

[4] Zhu L, Chen T, Xu L, Zhou Z, Feng W, Liu Y, Chen H. 2020. Effect and mechanism of quorum sensing on horizontal transfer of multidrug plasmid RP4 in BAC biofilm. Sci Total Environ 698:134236. doi:10.1016/j.scitotenv.2019.134236 31493577

[5] Rahbari KM, Chang JC, Federle MJ. 2021. A *Streptococcus* quorum sensing system enables suppression of innate immunity. mBio 12:e03400-20. doi:10.1128/mBio.03400-20 PMC8262891 33947757

[6] Moreno-Gámez S, Hochberg ME, van Doorn GS. 2023. Quorum sensing as a mechanism to harness the wisdom of the crowds. Nat Commun 14:3415. doi:10.1038/s41467-023-37950-7 PMC10256802 37296108

[7] Jiang K, Xu Y, Yuan B, Yue Y, Zhao M, Luo R, Wu H, Wang L, Zhang Y, Xiao J, Lin F. 2022. Effect of autoinducer-2 quorum sensing inhibitor on interspecies quorum sensing. Front Microbiol 13:791802. doi:10.3389/fmicb.2022.791802 PMC8996156 35418956

[8] Zhang L, Li S, Liu X, Wang Z, Jiang M, Wang R, Xie L, Liu Q, Xie X, Shang D, Li M, Wei Z, Wang Y, Fan C, Luo Z-Q, Shen X. 2020. Sensing of autoinducer-2 by functionally distinct receptors in prokaryotes. Nat Commun 11:5371. doi:10.1038/s41467-020-19243-5 PMC7584622 33097715

[9] Oliveira RA, Cabral V, Torcato I, Xavier KB. 2023. Deciphering the quorum-sensing lexicon of the gut microbiota. Cell Host Microbe 31:500–512. doi:10.1016/j.chom.2023.03.015 37054672

[10] Williams P, Cámara M. 2009. Quorum sensing and environmental adaptation in *Pseudomonas aeruginosa*: a tale of regulatory networks and multifunctional signal molecules. Curr Opin Microbiol 12:182–191. doi:10.1016/j.mib.2009.01.005 19249239

[11] Li Q, Liu L, Guo A, Zhang X, Liu W, Ruan Y. 2021. Formation of multispecies biofilms and their resistance to disinfectants in food processing environments: a review. J Food Prot 84:2071–2083. doi:10.4315/JFP-21-071 34324690

[12] Li Y-H, Tian X. 2012. Quorum sensing and bacterial social interactions in biofilms. Sensors 12:2519–2538. doi:10.3390/s120302519 PMC3376616 22736963

[13] Valente RS, Nadal-Jimenez P, Carvalho AFP, Vieira FJD, Xavier KB. 2017. Signal integration in quorum sensing enables cross-species induction of virulence in *Pectobacterium wasabiae*. mBio 8:e00398-17. doi:10.1128/mBio.00398-17 PMC5442451 28536283

[14] de Kievit TR, Iglewski BH. 2000. Bacterial quorum sensing in pathogenic relationships. Infect Immun 68:4839–4849. doi:10.1128/IAI.68.9.4839-4849.2000 PMC101676 10948095

[15] Twomey KB, O’Connell OJ, McCarthy Y, Dow JM, O’Toole GA, Plant BJ, Ryan RP. 2012. Bacterial *cis*-2-unsaturated fatty acids found in the cystic fibrosis airway modulate virulence and persistence of *Pseudomonas aeruginosa*. ISME J 6:939–950. doi:10.1038/ismej.2011.167 PMC3329111 22134647

[16] Markus V, Paul AA, Teralı K, Özer N, Marks RS, Golberg K, Kushmaro A. 2023. Conversations in the gut: the role of quorum sensing in normobiosis. Int J Mol Sci 24:3722. doi:10.3390/ijms24043722 PMC9963693 36835135

[17] de Rossi BP, García C, Alcaraz E, Franco M. 2014. *Stenotrophomonas maltophilia* interferes via the DSF-mediated quorum sensing system with *Candida albicans* filamentation and its planktonic and biofilm modes of growth. Rev Argent Microbiol 46:288–297. doi:10.1016/S0325-7541(14)70084-7 25576410

[18] Bellenberg S, Salas B, Ganji S, Jorquera-Román C, Valenzuela ML, Buetti-Dinh A, Unelius CR, Dopson M, Vera M. 2021. Diffusible signal factor signaling controls bioleaching activity and niche protection in the acidophilic, mineral-oxidizing leptospirilli. Sci Rep 11:16275. doi:10.1038/s41598-021-95324-9 PMC8357829 34381075

[19] Davies DG, Marques CNH. 2009. A fatty acid messenger is responsible for inducing dispersion in microbial biofilms. J Bacteriol 191:1393–1403. doi:10.1128/JB.01214-08 PMC2648214 19074399

[20] Boon C, Deng Y, Wang L-H, He Y, Xu J-L, Fan Y, Pan SQ, Zhang L-H. 2008. A novel DSF-like signal from *Burkholderia cenocepacia* interferes with *Candida albicans* morphological transition. ISME J 2:27–36. doi:10.1038/ismej.2007.76 18049456

[21] Tran TM, Ma Z, Triebl A, Nath S, Cheng Y, Gong B-Q, Han X, Wang J, Li J-F, Wenk MR, Torta F, Mayor S, Yang L, Miao Y. 2020. The bacterial quorum sensing signal DSF hijacks *Arabidopsis thaliana* sterol biosynthesis to suppress plant innate immunity. Life Sci Alliance 3:e202000720. doi:10.26508/lsa.202000720 PMC7425213 32788227

[22] Ma Z, Liu X, Nath S, Sun H, Tran TM, Yang L, Mayor S, Miao Y. 2021. Formin nanoclustering-mediated actin assembly during plant flagellin and DSF signaling. Cell Rep 34:108884. doi:10.1016/j.celrep.2021.108884 33789103

[23] He Y-W, Deng Y, Miao Y, Chatterjee S, Tran TM, Tian J, Lindow S. 2023. DSF-family quorum sensing signal-mediated intraspecies, interspecies, and inter-kingdom communication. Trends Microbiol 31:36–50. doi:10.1016/j.tim.2022.07.006 35941062

[24] Zhu H, Wang Z, Wang W, Lu Y, He Y-W, Tian J. 2022. Bacterial quorum-sensing signal DSF inhibits LPS-induced inflammations by suppressing Toll-like receptor signaling and preventing lysosome-mediated apoptosis in zebrafish. Int J Mol Sci 23:7110. doi:10.3390/ijms23137110 PMC9266882 35806111

[25] Ryan RP, An S, Allan JH, McCarthy Y, Dow JM. 2015. The DSF family of cell–cell signals: an expanding class of bacterial virulence regulators. PLoS Pathog 11:e1004986. doi:10.1371/journal.ppat.1004986 PMC4504480 26181439

[26] Barber CE, Tang JL, Feng JX, Pan MQ, Wilson TJG, Slater H, Dow JM, Williams P, Daniels MJ. 1997. A novel regulatory system required for pathogenicity of *Xanthomonas campestris* is mediated by a small diffusible signal molecule. Mol Microbiol 24:555–566. doi:10.1046/j.1365-2958.1997.3721736.x 9179849

[27] Wang L-H, He Y, Gao Y, Wu JE, Dong Y-H, He C, Wang SX, Weng L-X, Xu J-L, Tay L, Fang RX, Zhang L-H. 2004. A bacterial cell–cell communication signal with cross-kingdom structural analogues. Mol Microbiol 51:903–912. doi:10.1046/j.1365-2958.2003.03883.x 14731288

[28] Bi H, Christensen QH, Feng Y, Wang H, Cronan JE. 2012. The *Burkholderia cenocepacia* BDSF quorum sensing fatty acid is synthesized by a bifunctional crotonase homologue having both dehydratase and thioesterase activities. Mol Microbiol 83:840–855. doi:10.1111/j.1365-2958.2012.07968.x PMC3276249 22221091

[29] He Y-W, Wang C, Zhou L, Song H, Dow JM, Zhang L-H. 2006. Dual signaling functions of the hybrid sensor kinase RpfC of *Xanthomonas campestris* involve either phosphorelay or receiver domain-protein interaction. J Biol Chem 281:33414–33421. doi:10.1074/jbc.M606571200 16940295

[30] Huang T-P, Lee Wong AC. 2007. Extracellular fatty acids facilitate flagella-independent translocation by *Stenotrophomonas maltophilia*. Res Microbiol 158:702–711. doi:10.1016/j.resmic.2007.09.002 18054205

[31] Beaulieu ED, Ionescu M, Chatterjee S, Yokota K, Trauner D, Lindow S. 2013. Characterization of a diffusible signaling factor from *Xylella fastidiosa*. mBio 4:e00539-12. doi:10.1128/mBio.00539-12 PMC3546559 23300249

[32] Dow M. 2008. Diversification of the function of cell-to-cell signaling in regulation of virulence within plant pathogenic xanthomonads. Sci Signal 1:pe23. doi:10.1126/stke.121pe23 18506032

[33] Waldron EJ, Snyder D, Fernandez NL, Sileo E, Inoyama D, Freundlich JS, Waters CM, Cooper VS, Neiditch MB. 2019. Structural basis of DSF recognition by its receptor RpfR and its regulatory interaction with the DSF synthase RpfF. PLoS Biol 17:e3000123. doi:10.1371/journal.pbio.3000123 PMC6361424 30716063

[34] Suppiger A, Eshwar AK, Stephan R, Kaever V, Eberl L, Lehner A. 2016. The DSF type quorum sensing signalling system RpfF/R regulates diverse phenotypes in the opportunistic pathogen *Cronobacter*. Sci Rep 6:18753. doi:10.1038/srep18753 PMC4698668 26725701

[35] Amari DT, Marques CNH, Davies DG. 2013. The putative enoyl-coenzyme A hydratase DspI is required for production of the *Pseudomonas aeruginosa* biofilm dispersion autoinducer *cis*-2-decenoic acid. J Bacteriol 195:4600–4610. doi:10.1128/JB.00707-13 PMC3807434 23935049

[36] Huang J, Zhou T, Zhou X, Xue Y, Zhao W, Liao L, Chang C, Zhang L-H, Xu Z. 2026. Dual-functional quorum sensing signal synthases DspII and DspI coordinate virulence switch in *Pseudomonas aeruginosa*. Nat Commun 17:1926. doi:10.1038/s41467-026-68622-x PMC12923603 41571658

[37] Kalia M, Amari D, Davies DG, Sauer K. 2023. *cis*-DA-dependent dispersion by *Pseudomonas aeruginosa* biofilm and identification of *cis*-DA-sensory protein DspS. mBio 14:e02570-23. doi:10.1128/mbio.02570-23 PMC10746223 38014955

[38] Liu L, Li T, Cheng X-J, Peng C-T, Li C-C, He L-H, Ju S-M, Wang N-Y, Ye T-H, Lian M, Xiao Q-J, Song Y-J, Zhu Y-B, Yu L-T, Wang Z-L, Bao R. 2018. Structural and functional studies on *Pseudomonas aeruginosa* DspI: implications for its role in DSF biosynthesis. Sci Rep 8:3928. doi:10.1038/s41598-018-22300-1 PMC5834635 29500457

[39] An S-Q, Murtagh J, Twomey KB, Gupta MK, O’Sullivan TP, Ingram R, Valvano MA, Tang J-L. 2019. Modulation of antibiotic sensitivity and biofilm formation in *Pseudomonas aeruginosa* by interspecies signal analogues. Nat Commun 10:2334. doi:10.1038/s41467-019-10271-4 PMC6536496 31133642

[40] Sorbara MT, Pamer EG. 2019. Interbacterial mechanisms of colonization resistance and the strategies pathogens use to overcome them. Mucosal Immunol 12:1–9. doi:10.1038/s41385-018-0053-0 PMC6312114 29988120

[41] J Worley M. 2023. Immune evasion and persistence in enteric bacterial pathogens. Gut Microbes 15:2163839. doi:10.1080/19490976.2022.2163839 PMC9833415 36617629

[42] Diard M, Garcia V, Maier L, Remus-Emsermann MNP, Regoes RR, Ackermann M, Hardt W-D. 2013. Stabilization of cooperative virulence by the expression of an avirulent phenotype. Nature 494:353–356. doi:10.1038/nature11913 23426324

[43] Sturm A, Heinemann M, Arnoldini M, Benecke A, Ackermann M, Benz M, Dormann J, Hardt W-D. 2011. The cost of virulence: retarded growth of *Salmonella* Typhimurium cells expressing type III secretion system 1. PLoS Pathog 7:e1002143. doi:10.1371/journal.ppat.1002143 PMC3145796 21829349

[44] Galán JE, Curtiss R. 1989. Cloning and molecular characterization of genes whose products allow *Salmonella* typhimurium to penetrate tissue culture cells. Proc Natl Acad Sci USA 86:6383–6387. doi:10.1073/pnas.86.16.6383 PMC297844 2548211

[45] Ellermeier CD, Ellermeier JR, Slauch JM. 2005. HilD, HilC and RtsA constitute a feed forward loop that controls expression of the SPI1 type three secretion system regulator *hilA* in *Salmonella enterica* serovar Typhimurium. Mol Microbiol 57:691–705. doi:10.1111/j.1365-2958.2005.04737.x 16045614

[46] Golubeva YA, Ellermeier JR, Cott Chubiz JE, Slauch JM. 2016. Intestinal long-chain fatty acids act as a direct signal to modulate expression of the salmonella pathogenicity island 1 type III secretion system. mBio 7:e02170-15. doi:10.1128/mBio.02170-15 PMC4752608 26884427

[47] Prouty AM, Gunn JS. 2000. *Salmonella enterica* serovar typhimurium invasion is repressed in the presence of bile. Infect Immun 68:6763–6769. doi:10.1128/IAI.68.12.6763-6769.2000 PMC97778 11083793

[48] Eade CR, Hung C-C, Bullard B, Gonzalez-Escobedo G, Gunn JS, Altier C. 2016. Bile acids function synergistically to repress invasion gene expression in salmonella by destabilizing the invasion regulator HilD. Infect Immun 84:2198–2208. doi:10.1128/IAI.00177-16 PMC4962646 27185788

[49] Bosire EM, Eade CR, Schiltz CJ, Reid AJ, Troutman J, Chappie JS, Altier C. 2020. Diffusible signal factors act through AraC-type transcriptional regulators as chemical cues to repress virulence of enteric pathogens. Infect Immun 88:e00226-20. doi:10.1128/IAI.00226-20 PMC7504960 32690633

[50] Hung C-C, Garner CD, Slauch JM, Dwyer ZW, Lawhon SD, Frye JG, McClelland M, Ahmer BMM, Altier C. 2013. The intestinal fatty acid propionate inhibits *Salmonella* invasion through the post-translational control of HilD. Mol Microbiol 87:1045–1060. doi:10.1111/mmi.12149 PMC3581741 23289537

[51] Chowdhury R, Pavinski Bitar PD, Adams MC, Chappie JS, Altier C. 2021. AraC-type regulators HilC and RtsA are directly controlled by an intestinal fatty acid to regulate *Salmonella* invasion. Mol Microbiol 116:1464–1475. doi:10.1111/mmi.14835 PMC8688230 34687258

[52] Bosire EM, Adams MC, Pavinski Bitar PD, Murphy SG, Shin J-H, Chappie JS, Dörr T, Altier C. 2025. *Vibrio cholerae* integrates interspecies quorum-sensing signals to regulate virulence. mBio 16:e01537-25. doi:10.1128/mbio.01537-25 PMC12421807 40742133

[53] Chowdhury R, Bitar PDP, Bell KE, Altier C. 2023. *Shigella flexneri* utilizes intestinal signals to control its virulence. Gut Microbes 15:2256767. doi:10.1080/19490976.2023.2256767 PMC10519361 37741806

[54] Chowdhury R, Pavinski Bitar PD, Keresztes I, Condo AM, Altier C. 2021. A diffusible signal factor of the intestine dictates *Salmonella* invasion through its direct control of the virulence activator HilD. PLoS Pathog 17:e1009357. doi:10.1371/journal.ppat.1009357 PMC7932555 33617591

[55] Holý O, Forsythe S. 2014. *Cronobacter* spp. as emerging causes of healthcare-associated infection. J Hosp Infect 86:169–177. doi:10.1016/j.jhin.2013.09.011 24332367

[56] Forsythe SJ. 2018. Updates on the *Cronobacter* genus. Annu Rev Food Sci Technol 9:23–44. doi:10.1146/annurev-food-030117-012246 29272187

[57] Moreira de Gouveia MI, Bernalier-Donadille A, Jubelin G. 2024. *Enterobacteriaceae* in the human gut: dynamics and ecological roles in health and disease. Biology (Basel) 13:142. doi:10.3390/biology13030142 PMC10967970 38534413

[58] Yin Q, da Silva AC, Zorrilla F, Almeida AS, Patil KR, Almeida A. 2025. Ecological dynamics of Enterobacteriaceae in the human gut microbiome across global populations. Nat Microbiol 10:541–553. doi:10.1038/s41564-024-01912-6 PMC11790488 39794474

[59] Keller R, Pedroso MZ, Ritchmann R, Silva RM. 1998. Occurrence of virulence-associated properties in *Enterobacter cloacae*. Infect Immun 66:645–649. doi:10.1128/IAI.66.2.645-649.1998 PMC113501 9453621

[60] Martinson JNV, Pinkham NV, Peters GW, Cho H, Heng J, Rauch M, Broadaway SC, Walk ST. 2019. Rethinking gut microbiome residency and the *Enterobacteriaceae* in healthy human adults. ISME J 13:2306–2318. doi:10.1038/s41396-019-0435-7 PMC6776003 31089259

[61] Higgins DE, DiRita VJ. 1994. Transcriptional control of *toxT*, a regulatory gene in the ToxR regulon of *Vibrio cholerae*. Mol Microbiol 14:17–29. doi:10.1111/j.1365-2958.1994.tb01263.x 7830555

[62] Inhülsen S, Aguilar C, Schmid N, Suppiger A, Riedel K, Eberl L. 2012. Identification of functions linking quorum sensing with biofilm formation in *Burkholderia cenocepacia* H111. Microbiologyopen 1:225–242. doi:10.1002/mbo3.24 PMC3426421 22950027

[63] Barraud N, Kjelleberg S, Rice SA. 2015. Dispersal from microbial biofilms. Microbiol Spectr 3. doi:10.1128/microbiolspec.MB-0015-2014 27337281

[64] Alcaraz E, García C, Friedman L, de Rossi BP. 2019. The *rpf*/DSF signalling system of *Stenotrophomonas maltophilia* positively regulates biofilm formation, production of virulence-associated factors and β-lactamase induction. FEMS Microbiol Lett 366:fnz069. doi:10.1093/femsle/fnz069 31044250

[65] Rai R, Ranjan M, Pradhan BB, Chatterjee S. 2012. Atypical regulation of virulence-associated functions by a diffusible signal factor in *Xanthomonas oryzae* pv. *oryzae*. Mol Plant Microbe Interact 25:789–801. doi:10.1094/MPMI-11-11-0285-R 22352717

[66] Torres PS, Malamud F, Rigano LA, Russo DM, Marano MR, Castagnaro AP, Zorreguieta A, Bouarab K, Dow JM, Vojnov AA. 2007. Controlled synthesis of the DSF cell–cell signal is required for biofilm formation and virulence in *Xanthomonas campestris*. Environ Microbiol 9:2101–2109. doi:10.1111/j.1462-2920.2007.01332.x PMC1974818 17635553

[67] Yang Y, Xu B, Xu R, Xu G, Chen L, Huang S, Shi X, Ng HY, Lee C-H. 2026. Switch-like effects and regulatory mechanisms of DSF-mediated quorum sensing in wastewater biofilm behaviors. Water Res 300:125915. doi:10.1016/j.watres.2026.125915 42001570

[68] Lindenberg S, Klauck G, Pesavento C, Klauck E, Hengge R. 2013. The EAL domain protein YciR acts as a trigger enzyme in a c-di-GMP signalling cascade in *E. coli* biofilm control. EMBO J 32:2001–2014. doi:10.1038/emboj.2013.120 PMC3715855 23708798

[69] Serra DO, Hengge R. 2019. A c-di-GMP-based switch controls local heterogeneity of extracellular matrix synthesis which is crucial for integrity and morphogenesis of *Escherichia coli* macrocolony biofilms. J Mol Biol 431:4775–4793. doi:10.1016/j.jmb.2019.04.001 30954572

[70] Lamprecht O, Ratnikava M, Jacek P, Kaganovitch E, Buettner N, Fritz K, Biazruchka I, Köhler R, Pietsch J, Sourjik V. 2023. Regulation by cyclic di-GMP attenuates dynamics and enhances robustness of bimodal curli gene activation in *Escherichia coli*. PLoS Genet 19:e1010750. doi:10.1371/journal.pgen.1010750 PMC10212085 37186613

[71] Chowdhury R, Pavinski Bitar PD, Chapman HM, Altier C. 2023. *Salmonella* invasion is controlled by competition among intestinal chemical signals. mBio 14:e00012-23. doi:10.1128/mbio.00012-23 PMC10127606 37017539

[72] Katoh K, Misawa K, Kuma K, Miyata T. 2002. MAFFT: a novel method for rapid multiple sequence alignment based on fast Fourier transform. Nucleic Acids Res 30:3059–3066. doi:10.1093/nar/gkf436 PMC135756 12136088

[73] Quast C, Pruesse E, Yilmaz P, Gerken J, Schweer T, Yarza P, Peplies J, Glöckner FO. 2013. The SILVA ribosomal RNA gene database project: improved data processing and web-based tools. Nucleic Acids Res 41:D590–D596. doi:10.1093/nar/gks1219 PMC3531112 23193283

[74] Pages H, Aboyoun P, Gentleman R, DebRoy S. 2026. Biostrings: efficient manipulation of biological strings (R package version 2.81.1).

[75] Minh BQ, Schmidt HA, Chernomor O, Schrempf D, Woodhams MD, von Haeseler A, Lanfear R. 2020. IQ-TREE 2: new models and efficient methods for phylogenetic inference in the genomic era. Mol Biol Evol 37:1530–1534. doi:10.1093/molbev/msaa015 PMC7182206 32011700

[76] Wong T, Ly-Trong N, Ren H, Baños H, Roger A, Susko E, Bielow C, De Maio N, Goldman N, Hahn M, Huttley G, Lanfear R, Minh BQ. 2025. IQ-TREE 3: phylogenomic inference software using complex evolutionary models. EcoEvoRxiv. doi:10.32942/X2P62N PMC13191116 42085559

[77] Letunic I, Bork P. 2007. Interactive Tree Of Life (iTOL): an online tool for phylogenetic tree display and annotation. Bioinformatics 23:127–128. doi:10.1093/bioinformatics/btl529 17050570

[78] Chang AC, Cohen SN. 1978. Construction and characterization of amplifiable multicopy DNA cloning vehicles derived from the P15A cryptic miniplasmid. J Bacteriol 134:1141–1156. doi:10.1128/jb.134.3.1141-1156.1978 PMC222365 149110

[79] Datsenko KA, Wanner BL. 2000. One-step inactivation of chromosomal genes in *Escherichia coli* K-12 using PCR products. Proc Natl Acad Sci USA 97:6640–6645. doi:10.1073/pnas.120163297 PMC18686 10829079

[80] Dole VP. 1956. A relation between non-esterified fatty acids in plasma and the metabolism of glucose. J Clin Invest 35:150–154. doi:10.1172/JCI103259 PMC438791 13286333

[81] Dole VP, Meinertz H. 1960. Microdetermination of long-chain fatty acids in plasma and tissues. J Biol Chem 235:2595–2599. doi:10.1016/S0021-9258(19)76920-8 13817286

[82] Iwanaga M, Yamamoto K. 1985. New medium for the production of cholera toxin by *Vibrio cholerae* O1 biotype El Tor. J Clin Microbiol 22:405–408. doi:10.1128/jcm.22.3.405-408.1985 PMC268420 2995438

[83] Elsinghorst EA. 1994. Measurement of invasion by gentamicin resistance. Methods Enzymal 236:405–420. doi:10.1016/0076-6879(94)36030-8 7968625

[84] O’Toole GA. 2011. Microtiter dish biofilm formation assay. J Vis Exp 2437:2437. doi:10.3791/2437 PMC3182663 21307833

[85] Partridge JD. 2022. Surveying a swarm: experimental techniques to establish and examine bacterial collective motion. Appl Environ Microbiol 88:e01853-21. doi:10.1128/AEM.01853-21 PMC8824199 34878816

