# Supplementary material for: Members of the Enterobacteriaceae family produce a diffusible signal factor that modulates enteric pathogen virulence
Source: mSphere. 2026 Oct 8;11(10):e00403-26. doi: 10.1128/msphere.00403-26 (PMC13711827; doi:10.1128/msphere.00403-26)
Supplement: Supplementary file 1 [file msphere.00403-26-s0002.pdf]

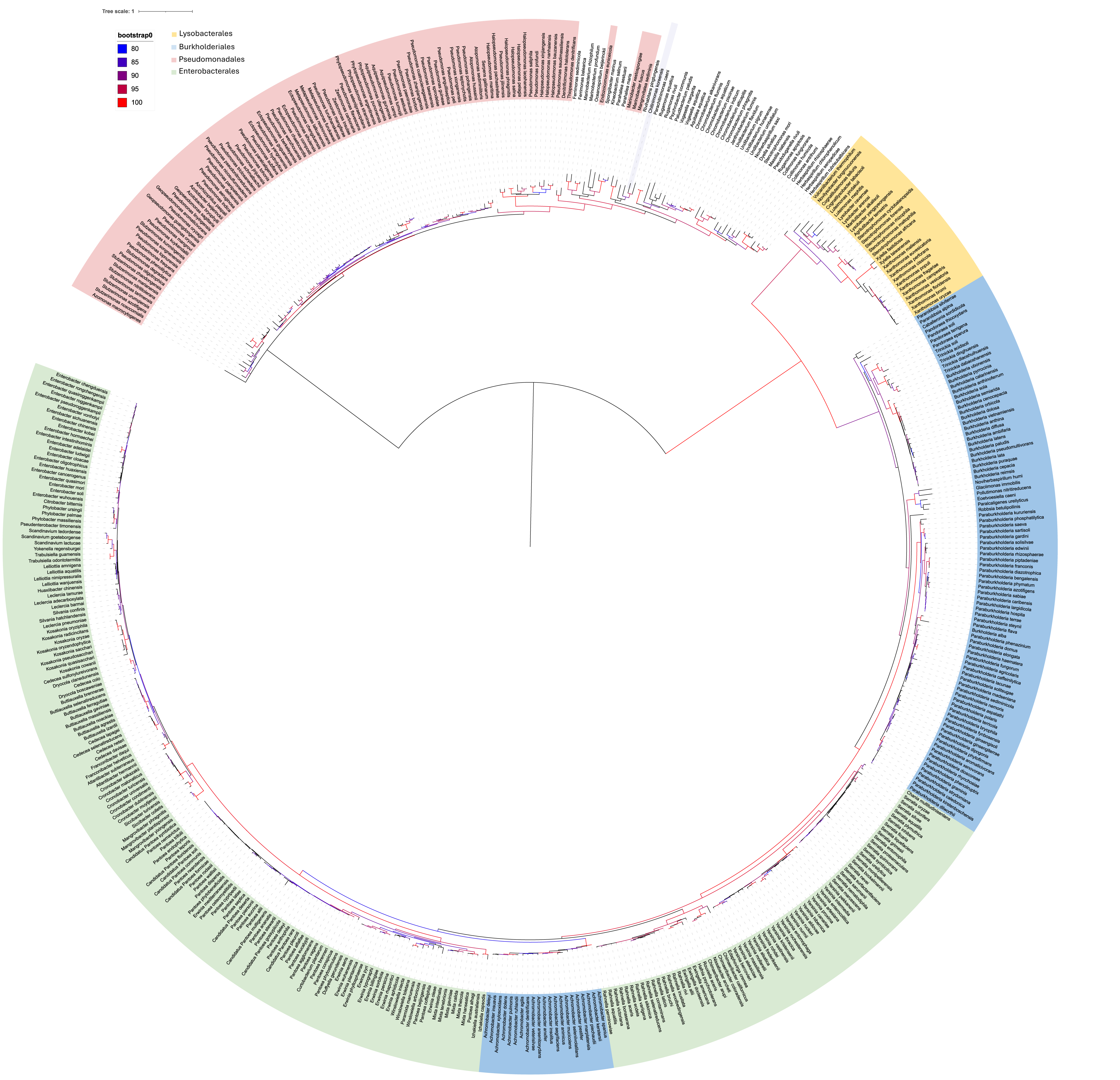
Tree scale: 1
bootstrap0
80
85
90
95
100
Lysobacterales
Burkholderiales
Pseudomonadales
Enterobacterales
