# Supplementary material for: Members of the Enterobacteriaceae family produce a diffusible signal factor that modulates enteric pathogen virulence
Source: mSphere. 2026 Oct 8;11(10):e00403-26. doi: 10.1128/msphere.00403-26 (PMC13711827; doi:10.1128/msphere.00403-26)
Supplement: Supplementary file 2 [file msphere.00403-26-s0003.pdf]

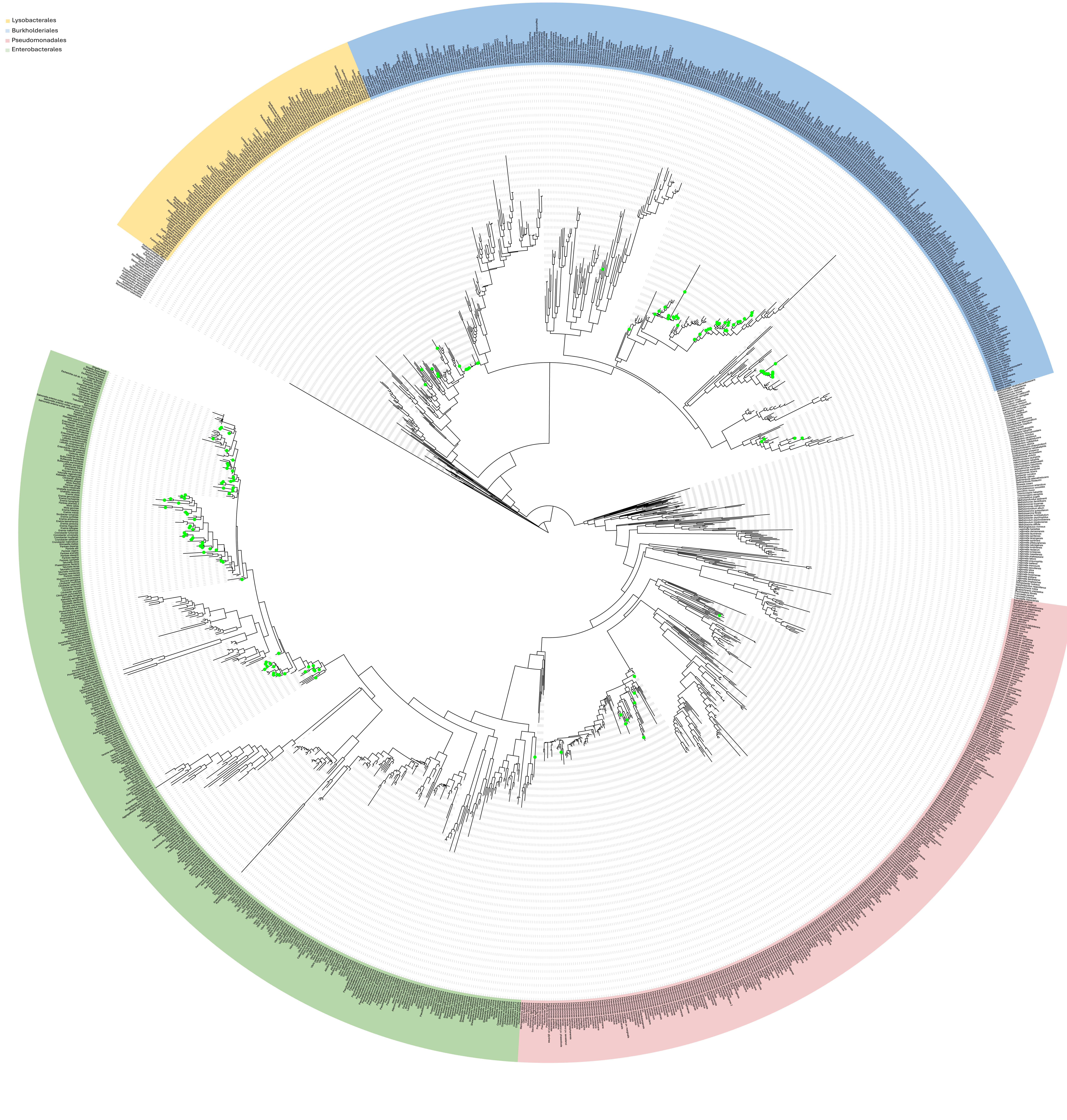
Lysobacterales
Burkholderiales
Pseudomonadales
Enterobacterales
