# Supplementary material for: Members of the Enterobacteriaceae family produce a diffusible signal factor that modulates enteric pathogen virulence
Source: mSphere. 2026 Oct 8;11(10):e00403-26. doi: 10.1128/msphere.00403-26 (PMC13711827; doi:10.1128/msphere.00403-26)
Supplement: Supplementary file 3 [file msphere.00403-26-s0004.docx]

**TITLE: Members of the *Enterobacteriaceae* family produce a diffusible signal factor that modulates enteric pathogen virulence**

Paulina D. Pavinski Bitar^1^, Rimi Chowdhury^2^, Erick Maosa Bosire^1^, Ivan Keresztes^3^, Michael J. Stanhope^4^, and Craig Altier^1^*

^1^ Department of Population Medicine and Diagnostic Sciences, College of Veterinary

Medicine, Cornell University, Ithaca, New York, USA

^2^ Department of Microbiology, College of Arts and Science, Miami University, Oxford, Ohio, USA

^3^ Department of Chemistry and Chemical Biology, Cornell University, Ithaca, New York, USA

^4^ Department of Public and Ecosystem Health, College of Veterinary Medicine, Cornell University, Ithaca, New York, USA

Correspondence: altier@cornell.edu

**Supplementary Legends**

**Figure S1: Homologues of the Diffusible Signal Factor synthase RpfF form three distinct clades.**

Maximum-likelihood phylogenetic tree constructed from 502 RpfF amino acid sequences. Three major clades are indicated based on phylogenetic topology. Taxonomic groups corresponding to *Lysobacterales*, *Pseudomonadales*, *Burkholderiales*, and *Enterobacterales* are color-coded as indicated. Schematic representations of *rpfF* operon architectures are shown adjacent to each clade. Branches with strong support (SH-like approximate likelihood ratio test >80%) are highlighted in blue.

**Figure S2: High resolution phylogenetic tree based on RpfF amino acid sequences.**

Maximum-likelihood phylogenetic tree constructed from 502 RpfF amino acid sequences. Three major clades are indicated based on phylogenetic topology. Taxonomic groups corresponding to *Lysobacterales*, *Pseudomonadales*, *Burkholderiales*, and *Enterobacterales* are color-coded as indicated. Branches with strong support (SH-like approximate likelihood ratio test >80%) are highlighted.

**Figure S3: RpfF homologue presence mapped onto a 16S rRNA phylogenetic tree of the Gammaproteobacteria**

Maximum-likelihood phylogenetic tree based on 16S rRNA gene sequences from representative Gammaproteobacteria. Taxonomic groups corresponding to *Lysobacterales*, *Burkholderiales*, *Pseudomonadales*, and *Enterobacterales* are indicated. Species encoding an RpfF homologue (≥65% amino acid identity and ≥95% query coverage to a characterized DSF synthases) are marked with green circles.
